# Morphology and property control of NiO nanostructures for supercapacitor applications

**DOI:** 10.1186/1556-276X-8-363

**Published:** 2013-08-23

**Authors:** Farrukh Iqbal Dar, Kevin Radakishna Moonooswamy, Mohammed Es-Souni

**Affiliations:** 1 Institute for Materials and Surface Technology (IMST), University of Applied Sciences Kiel, Kiel 24149, Germany

**Keywords:** NiO nanostructures, AAO-aided template synthesis, Electrodeposition, Supercapacitor

## Abstract

We process one-dimensional (1D) NiO nanostructures in anodized alumina templates starting from electrochemically deposited Ni nanotubes (NTs), and characterize their morphology-dependent supercapacitance behavior. The morphology of the 1D NiO nanostructures is controlled by the time of annealing at 450°C. After 25 min of annealing, the NTs start to close but maintain the tubular structure, and after a further 300 min of annealing time, the tubes are completely closed and nanorods (NRs) are formed. We show that the structures obtained are highly promising for supercapacitor applications; the performance of the NiO NT structure is with a specific capacitance of 2,093 F/g, the highest ever obtained for NiO, approaching the theoretical capacitance of this material. A suitable combination of nanocrystalline grain size and the high surface area akin to the tubular structure is responsible for this high performance. In contrast, the NiO NR structure is characterized by lower performance (797 F/g). A further attribute of the proposed structure is its high stability against galvanostatic charging-discharging cycling at high current densities, with almost no alteration to performance after 500 cycles.

## Background

Electrochemical capacitors that are also designated supercapacitors [1] derive their energy storage capacity from interaction between electrode and electrolyte at the interfacial region. Supercapacitors are currently a prominent area of research for energy storage devices as they have high power density, long cycling life, and short charging time [2-4]. Moreover, they have higher energy density than conventional dielectric capacitors [1,4]. Supercapacitors can be used either alone as a primary power source or as an auxiliary one with rechargeable batteries for high-power applications, such as industrial mobile equipment and hybrid/electric vehicles.

Electrochemical capacitors can be further divided into two categories based on energy storage modes, that is, electrical double layer capacitors and redox or pseudocapacitors. In the former, charge separation takes place on either side of the interface leading to the formation of an electrochemical double layer. When a voltage is applied, a current is generated due to the rearrangement of charges [5,6]. Pseudocapacitors, in contrast, get their charge from the fast and reversible reduction and oxidation (redox) reaction that takes place at the electrode-electrolyte interface due to change in oxidation state [7-9]. These pseudocapacitors are characterized by superior capacitance compared to their double-layer counterparts [10].

A number of inorganic materials have been shown in the past to exhibit outstanding capacitor characteristics; among them, hydrous RuO_2_ showed the best performance, but its high cost limits its application as a supercapacitor [11,12]. Thus, the focus of the current research is being placed on low-cost materials such as NiO [13,14], MnO_2_[15], Ni(OH)_2_[16], Co_3_O_4_[17], and V_2_O_5_[18].

NiO-based nanostructures and thin films have been extensively applied as electrode materials for lithium-ion batteries and fuel cells [19-21], electrochromic films [22,23], gas sensors [24], and electrochemical supercapacitors [22,25]. Because NiO is cheaper than RuO_2_, environmentally benign, and easy to process using a variety of methods, it deserved, and continue to deserve, considerable research activities toward high-performance electrochemical supercapacitor applications [13,14,22,25,26].

A large specific surface area in redox energy storage supercapacitors ensures an efficient contact with more electroactive sites even at high current densities [26,27]. Efficient electrode-electrolyte contact also depends upon the kind of nanostructures and porosity [26,27]. Because the rate capability (charge–discharge) of the electrode materials is mainly determined by ion diffusion kinetics and electronic conductivity [28], nano/micro hierarchical porous superstructures are best suited as electrode materials in energy storage devices, especially one-dimensional (1D) nanostructures which provide short transport pathways for electrons and ions [29,30]. High-aspect-ratio and high-surface-area nanostructures provide easy diffusion paths and improved diffusivity, which is crucial for better performance, while low-aspect-ratio nanostructures provide good mechanical stability [31]. Thus, morphology plays a vital role in defining the performance of the supercapacitor electrode.

In the present work, we take advantage of anodized alumina (AAO) templates to process 1D NiO nanostructures starting from Ni nanotubes (NTs) that are oxidized to yield 1D NiO nanostructures. By judicious choice of annealing temperature and time, the morphology of NiO could be tuned from NTs to nanorods (NRs), thus allowing the investigation of morphological effects on energy storage capability. The results indeed show that NiO NTs are characterized by superior capacitance performance characteristics in comparison to NiO NRs.

## Methods

The following chemicals were used as purchased: nickel chloride (NiCl_2_·6H_2_O), nickel sulfate (NiSO_4_·7H_2_O), and boric acid (H_3_BO_3_) (Sigma-Aldrich, Munich, Germany) and NaOH (Roth, Karlsruhe, Germany). All the chemicals were of analytical grade purity. Deionized water was used to prepare aqueous solutions (≥18 MΩ). Commercial AAO templates (60 μm thick) were obtained from Whatman International (Kent, UK) with 200-nm pore size (although the actual pore size ranges from 220 to 280 nm). The electrochemical experiments were performed at room temperature in a standard three-electrode cell. The electrodeposition and cyclic voltammograms (CVs) were made using an electrochemical workstation (ZAHNER IM6e, Kronach, Germany), and charging-discharging tests were performed using Source Meter 2400 (Keithley, Cleveland, OH, USA). A Pt mesh and hydroflex (H_2_ reference electrode) were used as counter and reference electrodes, respectively. All potentials are referred to the standard hydrogen electrode (SHE).

The microstructure and morphology of the nanostructures were characterized with a high-resolution scanning electron microscope (Ultra Plus, Zeiss, Oberkochen, Germany). X-ray diffraction (X'Pert Pro system, PANalytical, Almelo, The Netherlands) data was obtained in grazing incident geometry with fixed angles of 1.5° and 0.05° step using monochromatic Cu Kα radiation ((*λ* = 1.5418*Å*)). The process steps for preparing the nanostructures were detailed in our previous paper [32] and are described briefly below.

One side of the AAO template was sputtered with 20-nm gold (Au) to make it conductive. Subsequently, a thin Ni layer was electroplated from an electrolyte containing 310 g/L NiSO_4_·7H_2_O, 50 g/L NiCl_2_·6H_2_O, and 40 g/L H_3_BO_3_ on the sputtered Au to close the AAO template completely. The supporting Ni layer was 350 nm thick. Then Ni nanotubes (Ni NTs) were grown electrochemically via a bottom-up approach from the same electrolyte (310 g/L NiSO_4_·7H_2_O, 50 g/L NiCl_2_·6H_2_O, and 40 g/L H_3_BO_3_) under potentiostatic conditions at −0.9 V for 50 s. These AAO templates containing Ni NT were washed several times with distilled water and dried in air. Several Ni NT samples were prepared by the procedure described above, and out of these three cracks, free samples (samples 1, 2, and 3) were selected for electrochemical experiments.

Sample 1 was not annealed while samples 2 and 3 were annealed in air within the AAO template from room temperature to 450°C (heating rate 400 K/h) and were kept at this temperature for 25 min (sample 2) and 300 min (sample 3), respectively. These annealed samples were taken out of the furnace and cooled down in air. All the three samples were glued with (non-conductive) double-sided adhesion tape to the SiO_2_ supporting substrate, before dissolving the AAO template with 5% NaOH. To estimate the maximum contribution of the supporting Ni layer to capacitance, a Ni film sample was prepared by electrodepositing Ni on an Au-sputtered SiO_2_ substrate under the same electrodeposition conditions and annealed at 450°C.

To measure the pseuodocapacitance of the electrodes, CVs were recorded in an aqueous electrolyte containing 1 M KOH between 0.35 and 0.850 V at different scan rates. The charge–discharge behavior at different current densities and long-term cycling stability were tested in 1 M KOH. Before each electrochemical experiment, N_2_ was bubbled in the electrolyte for 15 min. The electrochemical experiments were conducted on a minimum of three to five samples each.

## Results and discussion

**Figure 1:**
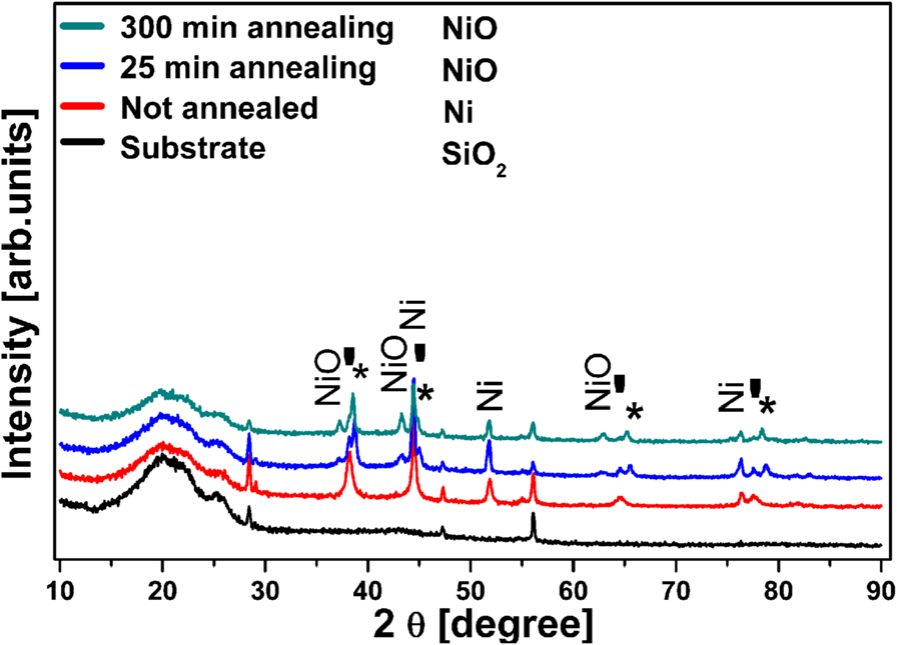
**XRD patterns of the non-annealed (sample 1) and annealed samples (samples 2 and 3).** The patterns are shifted vertically for clarity. The annealed samples show the presence of NiO peaks. The reflexes of Ni are still observed and arise from the incomplete oxidation of the Ni supporting layer. The stars and tick marks denote the Au-Ni alloy and Au, respectively.

The X-ray diffraction (XRD) patterns of the Ni (non-annealed sample 1) and NiO (annealed samples 2 and 3) nanostructures obtained under the deposition and annealing conditions described above are displayed in Figure 1. For the NiO nanostructures (samples 2 and 3), the NiO (cubic, NaCl structure) peaks become more distinguishable with increased annealing time. This is due to increasing oxide thickness along with enhanced crystal orientation. Using the Scherrer equation and the (200) reflection at 43.3°, the mean grain size calculated for sample 2 is 12.8 and that for sample 3 is 19.4 nm. The peaks indicated by a star (*) correspond to a Au-Ni binary alloy which is formed at this annealing temperature (450°C) due to the presence of sputtered Au. The chemical composition of this alloy was estimated from the peak positions, applying Vegard's law and using the lattice constants of *a* = 4.0789 Å for Au and *a* = 3.5238 Å for Ni. According to it, the Au-Ni alloy is composed of 90 at.% Au and 10 at.% Ni for the 25-min-annealed sample and 93 at.% Au and 7 at.% Ni for the 300-min-annealed samples.

From the above, it can be seen that metallic Ni still dominate the XRD spectrum, and it appears necessary to estimate the magnitude of oxidation of the nanostructures. For doing this, we make use of the data published in [33] which shows that Ni oxidation follows a parabolic law in a wide range of temperature. Through extrapolation and taking into account the surface area of the 1D morphology involved (see calculation details in Additional file 1: S1), it can be shown that sample 2 consists of 60% NiO while sample 3 is completely oxidized. Using the same procedure, only a small fraction of oxide (0.37%) is calculated for the underlying Ni layer, which explains the dominance of the Ni peaks in the XRD patterns.

**Figure 2:**
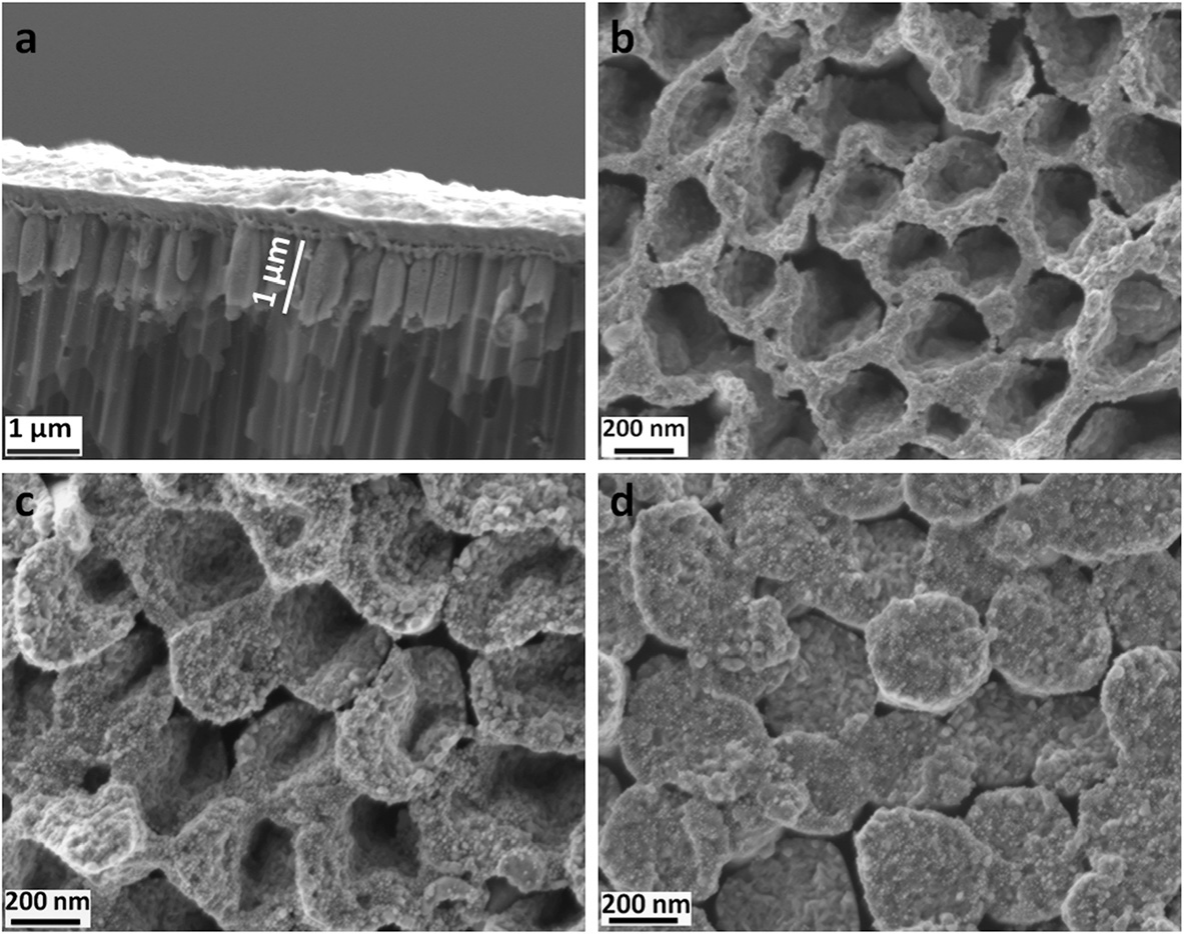
**SEM images of non-annealed (sample 1) and annealed samples (samples 2 and 3). (a)** Cross-sectional and **(b)** top views of the as-prepared Ni NT (non-annealed sample 1 inside AAO template). **(c)** Wall thickening after 25-min annealing (sample 2). **(d)** The complete closure of walls yielding NR morphology after 300-min annealing (sample 3).

The morphology of the nanostructures obtained is shown in Figure 2. The non-annealed sample 1 (Figure 2a, b) shows solely Ni NTs that form via nucleation and growth at the pore walls because of the presence of an extremely thin Au layer (see the experimental section and our previous paper [32]). The judicious deposition time for Ni to obtain NT is 50 s.

During annealing, the oxide layer nucleates and grows from the exposed inside walls and thickens in the direction of the inner-tube diameter. This suggests an outward diffusion of the Ni species toward oxygen ions. On the non-exposed outside walls that are confined by the AAO template, no oxide growth is expected. A short annealing time leads to incomplete oxidation of the Ni NTs, resulting in the formation of an oxide scale supported on a remaining Ni layer (see also the XRD results above and Additional file 1: S1). This is the case of sample 2 (Figure 2c; 25-min annealing). For longer annealing time, complete closure of the NT, to finally give the NR morphology as shown in Figure 2d, is achieved because of the volume increase associated with NiO oxide formation. This is the case of sample 3 (300-min annealing).

**Figure 3:**
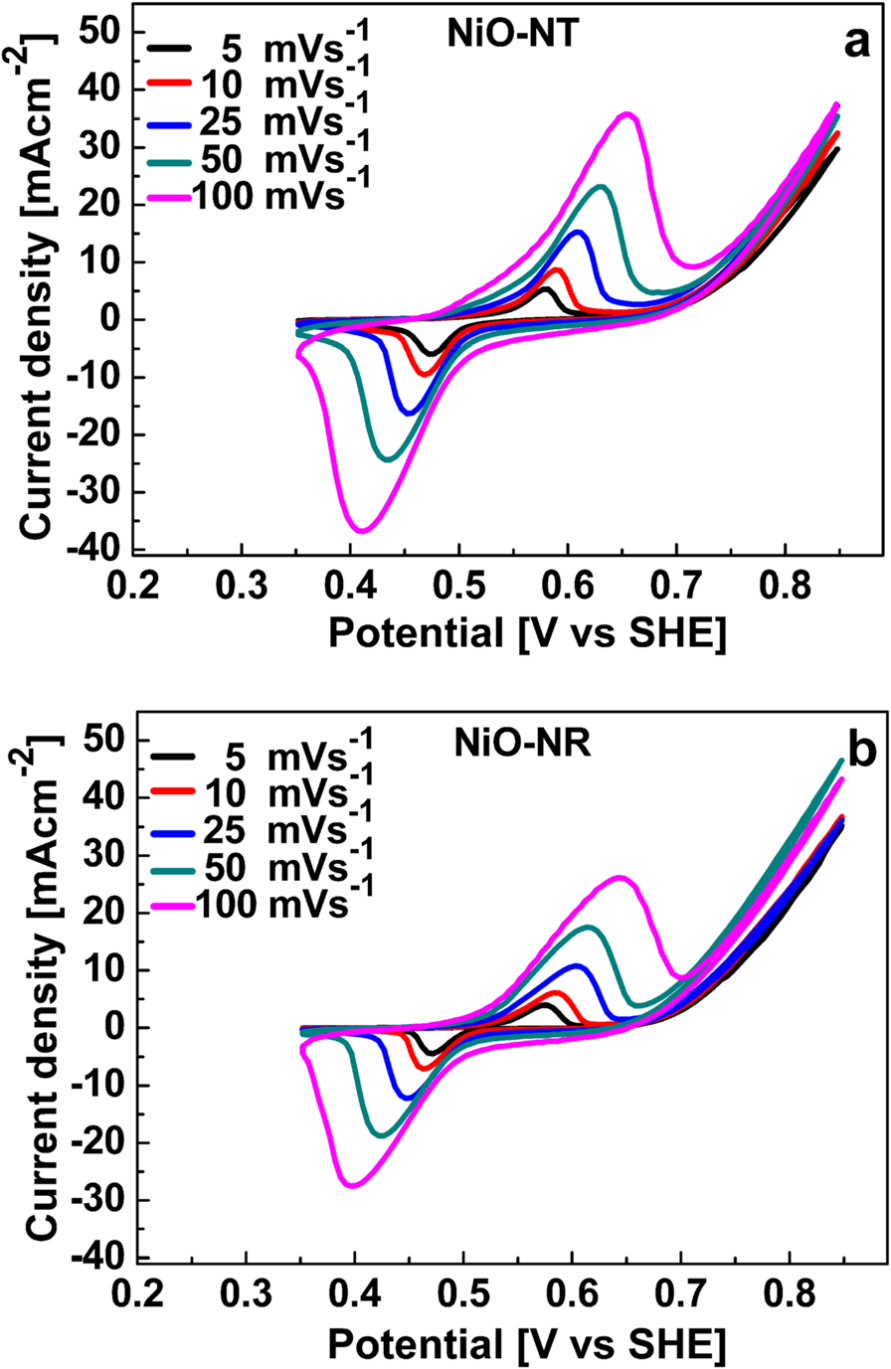
**CVs of nanostructures. (a)** NiO NT and **(b)** NiO NR electrodes in 1 M KOH at different scan rates in a potential window of 0.5 V.

Figure 3 shows the CV curves of the NiO NTs and NiO NRs recorded using a potential window of 0.5 V (between 0.35 and 0.85 V) at various scan rates (5, 10, 25, 50, and 100 mV/s). The two strong peaks observed in the anodic and cathodic directions correspond to the faradic redox reaction, expressed as follows [34,35]:

(1) $$\mathrm{NiO}+{\mathrm{OH}}^{-}↔\mathrm{NiOOH}+e^{-},$$

involving ionic and electronic transport at the NiO/electrolyte interface.

The shapes of the anodic and cathodic curves are similar for all scan rates. The profile of the CVs implies that the redox reaction at the interface of the nanostructure is reversible [36]. The peak current density increases with the scan rate because the redox reaction is diffusion-limited, and at a higher scan rate, the interfacial reaction kinetics and transport rate are not efficient enough. According to Equation 1, anions are exchanged with the electrolyte and electrode interface during redox reaction. This ion transfer process is slow and rate limiting, and higher scan rates are associated with smaller diffusion layer thickness [37]. This means that less of the electrode surface is utilized which lowers the resistivity and increases the current density that is also an indication of the pseudocapacitive behavior of the NiO nanostructures [36]. Further, the anodic and cathodic peaks are shifted to higher and lower potentials, respectively, with increasing scan rates (Figure 3). It again indicates that the ionic diffusion rate is not fast enough to keep pace with electronic neutralization in the redox reaction [38].

The specific capacitances were calculated from the CVs using the equation given below [39,40]:

(2) $$C=\frac{I}{2\cdot V\cdot S\cdot m},$$

where *C* is the specific capacitance (F/g), *I* the integrated area (V A) of the CV curve in one complete cycle, *V* the potential window (V), *S* the scan rate (V/s), and *m* the mass (g) of NiO, calculated using the oxidized Ni mass% outlined above, i.e., 60% and 100% for the NT and NR, respectively (Additional file 1: S1).

**Figure 4:**
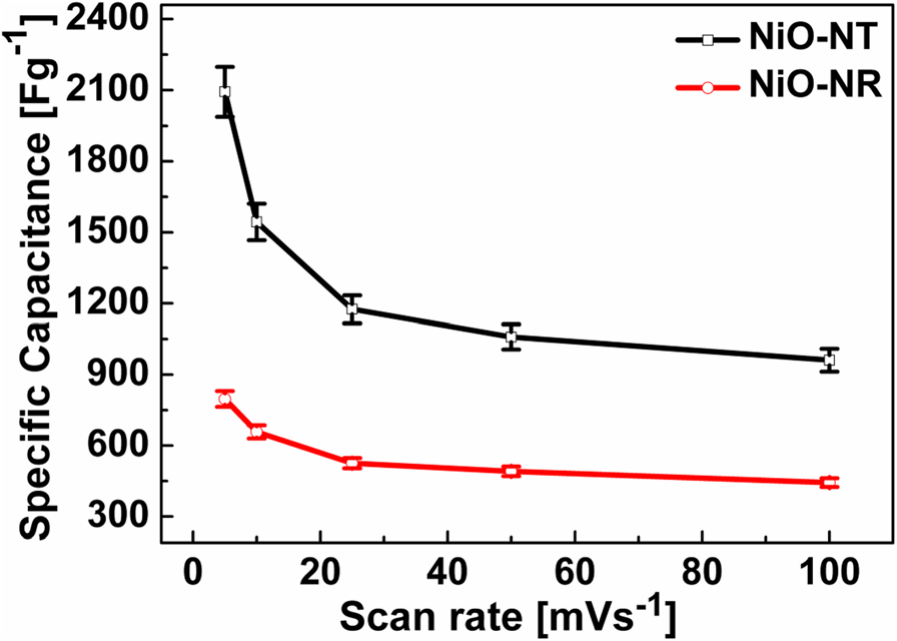
**The plot of the specific capacitance versus scan rate.** The dependence of the specific capacitance on the scan rate is shown for the NiO NT and NiO NR electrodes.

The dependence of the capacitance on the scan rate is depicted in Figure 4 and shows the downward trend with increasing scan rate discussed above. The error bars correspond to the standard deviation in mass, which is 5% (0.935 μg) and 4.2% (0.854 μg) for NiO NTs and NiO NRs, respectively.

Table 1 highlights the specific capacitances of our nanostructures and compares them with one of the recent works from the literature [14] at similar conditions of scan rates and electrolyte concentrations (1 M KOH). The specific values are for the capacitance obtained at slower scan rate because it represents nearly the full utilization of the electrode [41] through better ion penetration that is diffusion-limited [42]. Table 1 shows that the NiO NT sample is characterized by the highest specific capacitance (mean value of 2,093 F/g at 5 mV/s) while the NiO NR sample falls lower than the specific capacitance reported for NiO nanoporous films [14], except at 100 mV/s. The specific capacitance of the NiO NT sample is the highest ever reported for NiO nanostructures and approaches the theoretical value of approximately 2,584 F/g [43]. This is ascribed to the nanocrystalline nature of NiO grown in this work and the high surface area offered by the 1D NT nanostructure which ensures efficient contact with the electrolyte. We do not expect any contribution from NiO of the supporting layer for two reasons: firstly, only a negligible fraction of the Ni supporting layer is oxidized because the exposed area is very small due to the high density of the nanostructure, including the AAO template; secondly, even in the presence of an oxide layer, most of its area is occupied by the nanostructures and the effective exposed area (to the electrolyte) of the supporting layer is very small considering the average diameter (250 nm) and density (1 × 10^9^ cm^−2^) of the nanostructures. The maximum contribution of the underlying supporting NiO film was independently assessed on a plain Ni film of the same thickness, oxidized under the same conditions as above. The maximum capacitance was found to be 223 F/g at 5 mV/s scan rate (Additional file 1: Figure S2). This value of specific capacitance is for the fully utilized surface of the NiO film. This allows us to conclude that the capacitances measured reflect solely the contribution of our 1D nanostructures.

**Table 1:** Comparison of specific capacitances of different NiO nanostructures

**Scan rate (mV/s)**	**Specific capacitance (F/g)**		
	**NiO NR**	**NiO NT**	**NiO-nanoporous**
			**film [**[14]**]**
5	797	2,093	1,208
10	658	1,544	940
25	526	1,175	748
50	491	1,059	590
100	443	961	417

The NiO NT and NiO NR prepared in our work are compared with one of the recent works from the literature [14].

**Figure 5:**
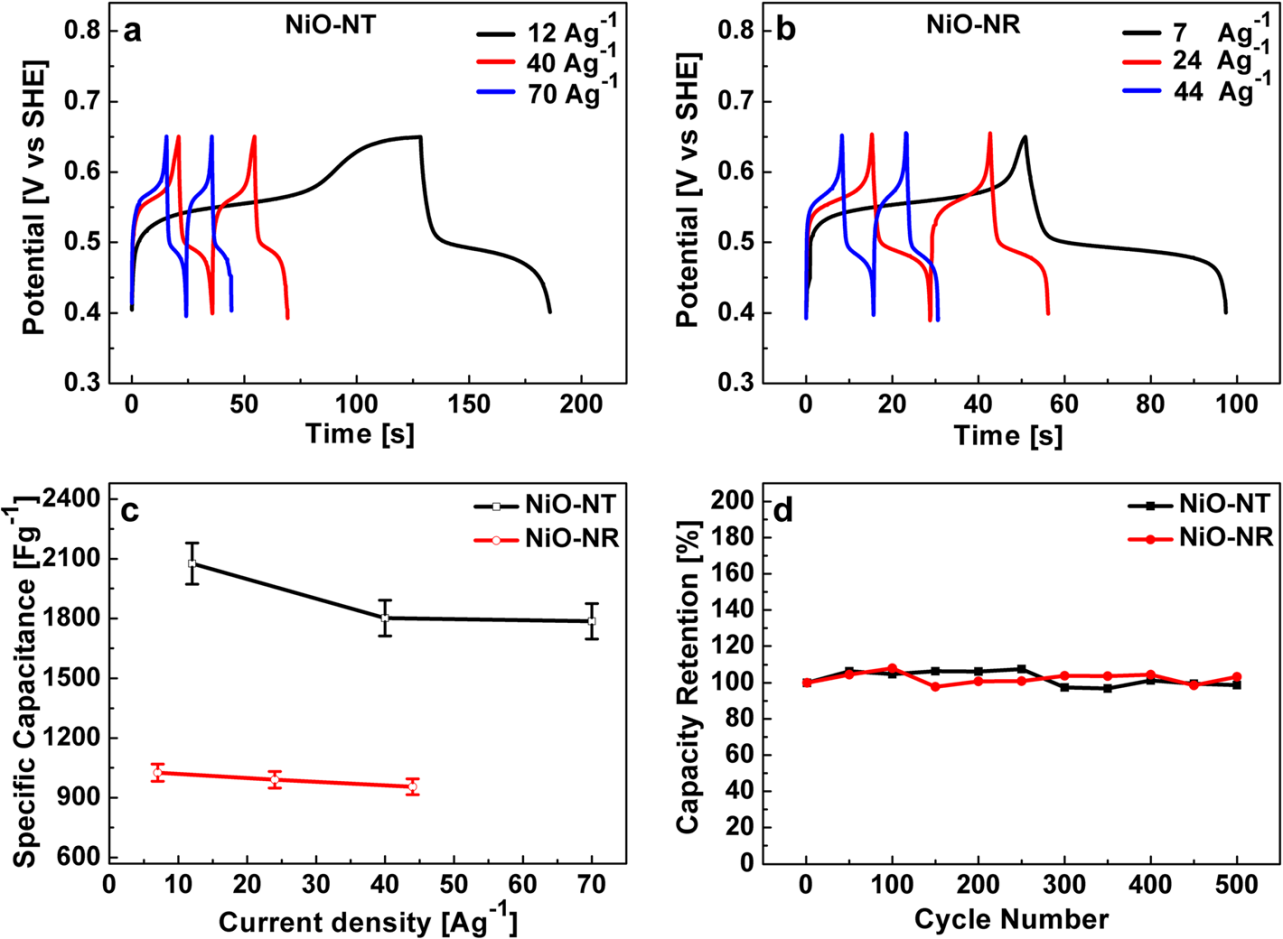
**The charge–discharge tests, rate capability, and long-term stability.** Charge–discharge tests of **(a)** NiO NT and **(b)** NiO NR electrodes in 1 M KOH at different constant current densities are shown. **(c)** Specific capacitance at different constant current densities shows the rate capability of NiO NT and NiO NR. **(d)** The capacity retention in a long-term cycling test (500 cycles) at a current density of 125 and 80 A/g for NiO NT and NiO NR, respectively. Both nanostructures show stable cycling performance.

The galvanostatic charging-discharging tests were performed at different constant current densities and are displayed in Figure 5a, b. The charge–discharge curves are non-linear with current density for both NiO nanostructures, as a further indication of their pseudocapacitive behavior [9].

From these charge–discharge curves, the specific capacitance was calculated at different current densities using the following equation:

(3) $$C=\frac{I\cdot t}{V\cdot m},$$

where *C* is the specific capacitance, *I* the current (A), *t* the discharge time (s), *m* the mass of NiO (g), and *V* the potential window (V). Figure 5c shows the specific capacitance as a function of current densities, which is the measure of the rate capability [44]. The specific capacitances of NiO NT are 2,076, 1,801, and 1,786 F/g at 12, 40, and 70 A/g, respectively, which demonstrates the unusually high power density obtained at high discharge current densities: 86% of capacitance is retained at the discharge current density of 70 A/g. The specific capacitances for NiO NR are 1,026, 990, and 955 F/g at 7, 24, and 44 A/g, respectively, which implies that the NiO NR structure retains 93% of its capacitance.

The long-term stability against cyclic charging-discharging is another important property of a capacitor structure. Figure 5d shows the long-term cycling performance of both NiO nanostructures at a constant current density of 125 and 80 A/g for NiO NT and NiO NR, respectively. Capacity retention over 500 cycles is almost 100% for both NiO nanostructures. The properties obtained for our nanostructures are outstanding in all aspects regarding supercapacitor performance. The NiO NT structure surpasses the results published so far on NiO supercapacitors; the maximum specific capacitance values (at constant current densities) achieved for NiO nanostructures of different morphologies, e.g., nanofibers [45], nanoflowers [46], nanoflakes [13], porous structures [47], nanoporous films [14], and nanorod arrays [48], span the range between 336 and 2,018 F/g (the latter value has been reported for NiO NR arrays on Ni foam at the fairly low current density of 2.2 F/g and is largely different from the value obtained for our NiO NR because of different structural dimensions). As outlined above, the nanocrystalline grain size together with the high surface area of the tubular structure is responsible for the high performance of the NiO NT structure that ensures an intimate contact with the electrolyte, i.e., offering a large density of active sites for OH^−^ ions for the redox reaction. Furthermore, the robustness and chemical stability of the nanostructures reported here are responsible for their stability against cyclic charging-discharging.

## Conclusions

One-dimensional NiO nanostructures for energy storage applications are processed using a combination of AAO-aided template synthesis and annealing treatments. The judicious selection of annealing time and temperature enabled us to control the morphology of the NiO nanostructures, from nanotubes to nanorods. Our electrochemical capacitance results show a large dependence of capacitance on morphology of the nanostructures. Particularly, the NiO NT structure shows outstanding capacitance properties with a capacitance value that surpasses those published so far in the literature for different NiO nanostructures. Beyond the achieved high capacitance value, the rate capability (charge-discharge capacitance at high current density) is also outstanding. Concerning the long-term stability on cyclic charging-discharging, full capacity retention is achieved for both nanostructures over 500 cycles.

## Competing interests

The authors declare that they have no competing interests.

## Authors' contributions

FID carried out the synthesis and characterization. KRM improved the manuscript and participated in the studies. MES conceived, planned, and directed the research and made final corrections to the manuscript. All authors read and approved the final manuscript.

## Supplementary Material

Additional file 1 **Magnitude of oxidation and specific capacitance of the NiO film.** Magnitude of oxidation (S1): interpolation of experimental data from the literature to calculate the parabolic rate constant Log *K*_p_ (Figure S1) and calculation of weight gain per unit area *M* (Table S1). Specific capacitance of NiO-Film (S2): the specific capacitance of the supporting NiO film is measured at different scan rates (Figure S2) to estimate the maximum contribution of the supporting NiO film. Click here for file
