# Supplementary material for: Morphology and property control of NiO nanostructures for supercapacitor applications
Source: Nanoscale Res Lett. 2013 Aug 23;8(1):363. doi: 10.1186/1556-276X-8-363 (PMC3765375; doi:10.1186/1556-276X-8-363)
Supplement: Additional file 1 — Magnitude of oxidation and specific capacitance of the NiO film. Magnitude of oxidation (S1): interpolation of experimental data from the literature to calculate the parabolic rate constant Log Kp (Figure S1) and calculation of weight gain per unit area M (Table S1). Specific capacitance of NiO-Film (S2): the specific capacitance of the supporting NiO film is measured at different scan rates (Figure S2) to estimate the maximum contribution of the supporting NiO film. [file 1556-276X-8-363-S1.docx]

**Additional file 1**

**Journal: Nanoscale Research Letters**

**Morphology and Property Control of NiO Nanostructures for Supercapacitor Applications**

Farrukh Iqbal Dar^1^, Kevin Radakishna Moonooswamy^1^, and Mohammed Es-Souni^1^*

^1^Institute for Materials and Surface Technology (IMST), University of Applied Sciences Kiel, Kiel 24149, Germany. Fax: +4943121062660; Tel: +49 0431-210-2660

*E-mail: mohammed.es-souni@fh-kiel.de

**S1: Magnitude of Oxidation**

The Ni follows a parabolic growth law of oxidation which is expressed as follows:

$$M^{2}=K_{p}\times\Theta$$

Where *M* is the weight gain per unit area, **the time of exposure (s) and *K_p_* the parabolic rate constant usually given in g^2^ cm^-4^ s^-1^.

The magnitude of oxidation is estimated by extrapolating the oxidation data for high purity Ni (> 99.95%) given in reference 33 to our temperature, in Figure S1as follows:


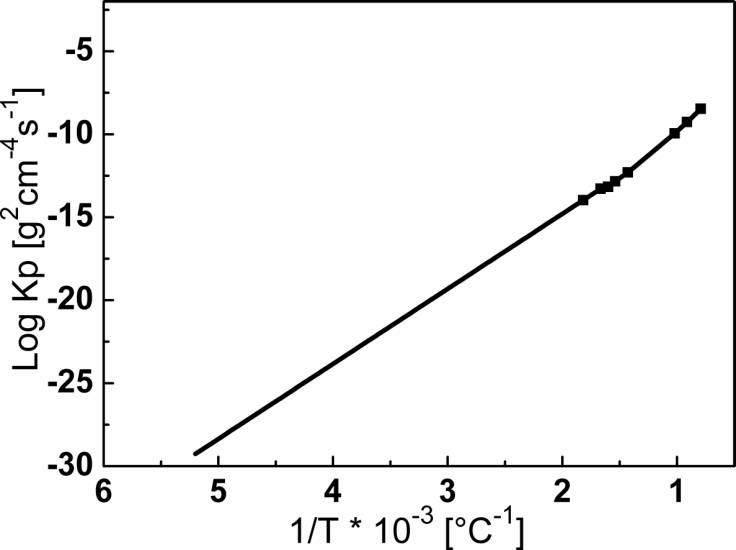


**Figure S1 The plot of Log K_p_ versus 1/T (T is temperature in °C)**. The K_p_ values for the temperature range from 1260 to 550 °C have been taken from reference 33 and we extrapolate them to 190 °C.

From this plot the K_p_ value at 450 °C is 1.616 × 10^-16^ g^2^cm^-4^s^-1^.

As sample 2 is oxidized for 1500 s (25 min) at 450 °C thus $K_{p}\times\Theta$ is 2.424 × 10^-13^ g^2^cm^-4^. And the value of *M* is 4.924 × 10^-7^ gcm^-2^.

Similarly, for sample 3, oxidized for 18000 s at the same temperature, the calculated value of *M* is 1.706 × 10^-6^ gcm^-2^.

Moreover, we also calculated the *M* values for temperature range from 200 to 445°C because we gradually heated the furnace at a heating rate of 400 K per hour to reach 450 °C after placing the samples in the furnace. For this purpose the *M* values are calculated at temperatures, 445, 440, 435, 430, 425, 420, 415, 410, 405, 400, 395, 390, 385, 380, 350, 320, 290, 250 and 200 °C for the corresponding times which is required to reach the next temperature as shown in Table S1.

**Table S1** **Weight gain per unit surface area (M) calculated by parabolic growth law at temperatures between 445 and 200 °C.**

| **Temperature** | **Time** | ***M*** |
| --- | --- | --- |
| [°C] | [s] | [gcm^-2^] |
| 445 | 45 | 8.445 × 10^-8^ |
| 440 | 45 | 6.864 × 10^-8^ |
| 435 | 45 | 6.786 × 10^-8^ |
| 430 | 45 | 5.328 × 10^-8^ |
| 425 | 45 | 4.2324 × 10^-8^ |
| 420 | 45 | 3.686 × 10^-8^ |
| 415 | 45 | 3.316 × 10^-8^ |
| 410 | 45 | 2.868 × 10^-8^ |
| 405 | 45 | 2.435 × 10^-8^ |
| 400 | 45 | 1.935 × 10^-8^ |
| 395 | 45 | 1.665 × 10^-8^ |
| 390 | 45 | 1.468 × 10^-8^ |
| 385 | 45 | 1.221 × 10^-8^ |
| 380 | 45 | 9.922 × 10^-9^ |
| 350 | 270 | 7.339 × 10^-9^ |
| 320 | 270 | 1.899 × 10^-9^ |
| 290 | 270 | 3.594 × 10^-10^ |
| 250 | 360 | 2.388 × 10^-11^ |
| 200 | 450 | 1.338 × 10^-13^ |

Below 200 °C the contribution of M values in oxidation is negligible. The sum of *M* values in the temperature range 445 to 200 °C (5.195 × 10^-7^ gcm^-2^) is added to the corresponding *M* value for oxidation at 450 °C for sample 2 and 3. Thus total calculated weight gain per unit area (M_c_) is 1.012 × 10^-6^ gcm^-2^ for sample 2 and 2.225 × 10^-6^ gcm^-2^ for sample 3.

The mass of Ni and surface area of both the samples described above were calculated to find the weight gain per unit area considering theoretical 100% oxidation (M_t_). The mass of Ni is calculated from the charge deposited during electrodeposition of nanostructures, thus mass of sample 2 is 18.551 × 10^-6^ g for 0.22 cm^2^ geometrical area (G) and sample 3, 16.141 × 10^-6^ g for 0.20 cm^2^ geometrical area.

The surface area (S) of the cylindrically shaped nanostructure electrode is calculated as follows:

S = (2πRh + 2πrh + 2πR^2^) × G × D

Where, D is the density of the AAO pores, 1 × 10^9^ cm^-2^, G is the geometrical area, R the outer radius of AAO pores, 125 nm (average outer diameter is 250 nm), r the inner radius, 75 nm (Initial average wall thickness of nanostructures after deposition is 50 nm, thus inner diameter is 150 nm) and h the average height of the nanostructures, 1000 nm.

The calculated S for sample 2 and 3 are 2.981 and 2.710 cm^2^ respectively. The weight of Ni per unit area is 6.223 × 10^-6^ gcm^-2^ for sample 2 and 5.956 × 10^-6^ gcm^-2^ for sample 3.

Assuming a 100 % oxidation of Ni in sample 2 and sample 3, calculated as follows:

Number of moles of NiO after oxidation = Number of moles of Ni before oxidation

$$Weight of NiO=\frac{weight of Ni \times Molecular weight of NiO \left( 74.71 g \mathrm{mole}^{-1} \right)}{Atomic weight of Ni \left( 58.71 g \mathrm{mole}^{-1} \right)}$$

the total weights of NiO in sample 2 and 3 are 23.607 × 10^-6^ and 20.540 × 10^-6^ g respectively and the weight gains for sample 2 and 3 are 5.056 × 10^-6^ and 4.399 × 10^-6^ g respectively for theoretical 100 % oxidation. It follows that the weight gain per unit surface area S should be 1.696 × 10^-6^ and 1.623 × 10^-6^ gcm^-2^ for sample 2 and 3, respectively. The above calculated value of M_c_ is 60% of M_t_ for sample 2 and M_c_ for sample 3 is higher than M_t_ which let us infer that it is completely oxidized. The mass of active material (NiO) is calculated from this percentage oxidation values. Thus masses of active material (NiO) in sample 2 and 3 are 14.131 and 20.539 µg respectively. Similarly, calculated NiO mass in supporting film is 15.655 µg.

**S2 Specific capacitance of NiO-Film**

**
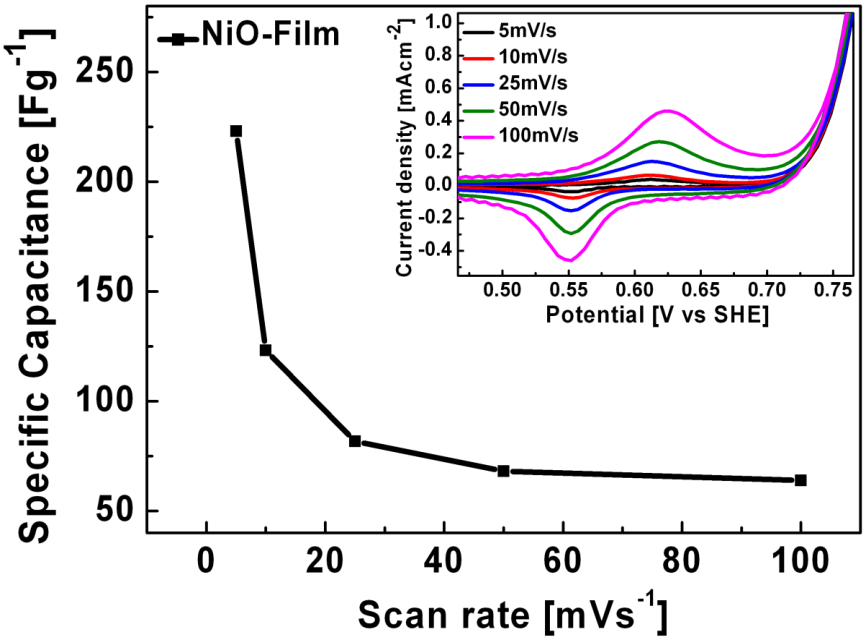
**

**Figure S2 Specific capacitance of the supporting NiO-film at different scan rates.** The inset shows the redox part of the CV curves. These specific capacitances were used to estimate the maximum contribution of the oxidation of the supporting layer, to the capacitance of the nanostructures. For this purpose a Ni layer was electrodeposited on Au sputtered SiO_2_ and subsequently annealed. The deposition annealing conditions were kept same as for the NiO-nanostructures.
